# The impact of supplementation with iso-branched chain fatty acids in a mouse model of atherosclerosis

**DOI:** 10.1007/s11033-025-11220-9

**Published:** 2025-11-12

**Authors:** Agata Zwara, Agata Jedrzejewska, Dorota Olszewska, Marta Tomczyk, Tomasz Sledzinski, Adriana Mika

**Affiliations:** 1 Department of Environmental Analytics, Faculty of Chemistry, University of Gdansk, Wita Stwosza 63, Gdansk, 80-308 Poland; 2 Department of Biochemistry, Faculty of Medicine, Medical University of Gdansk, Debinki 1, Gdansk, 80-211 Poland; 3 Department of Pharmaceutical Biochemistry, Faculty of Pharmacy, Medical University of Gdansk, Debinki 1, Gdansk, 80-211 Poland

**Keywords:** Atherosclerosis, Branched chain fatty acids, Triacylglycerols, CPT1, Metabolism

## Abstract

**Background:**

Serum iso-branched chain fatty acids (iso-BCFAs) levels are inversely correlated with triglyceride and high-sensitivity C-reactive protein (hs-CRP) concentration in obese patients. Moreover, they decrease the expression of genes related to lipogenesis and inflammation in hepatocytes. However, their role in atherosclerosis development remains unclear. This study aimed to assess the impact iso-BCFA supplementation on hyperlipidaemia and inflammation in atherosclerotic apolipoprotein E and low-density lipoprotein receptor double knockout (*ApoE*^*−/−*^*/Ldlr*^*⁻/⁻*^) mice.

**Methods:**

*ApoE*^*−/−*^*/Ldlr*^*⁻/⁻*^ mice were assigned to two experimental groups: (A) fed standard chow (SD-fed) and (B) fed iso-BCFA enriched (BCFA-fed) chow for 12 weeks. Blood samples were collected throughout the study, and tissues were harvested post-mortem. Biochemical analyses were performed using automated assays. FA composition was analyzed by gas chromatography-mass spectrometry (GC-MS). Atherosclerotic plaque area was assessed with Oil Red O staining and vascular inflammation via ecto-adenosine deaminase (eADA) activity. Hepatic gene expression was evaluated via real time PCR.

**Results:**

BCFA supplementation led to a stable increase in serum and tissue levels of these FAs. Significant reduction in serum triglyceride and total cholesterol (TC) concentrations was observed in the BCFA-fed group. No change in body weight or serum CRP was detected, whereas the mRNA levels of *Il6* in liver were lower after BCFA treatment. However, vascular lipid content and eADA activity were higher in BCFA-fed mice, and hepatic *Cpt1* mRNA expression significantly increased, suggesting enhanced β-oxidation.

**Conclusions:**

Dietary iso-BCFA supplementation may modulate lipid metabolism in *ApoE*^*−/−*^*/Ldlr*^*⁻/⁻*^mice. The reduction in triglycerides and TC suggests a potential antiatherosclerosis effects, whereas increased aorta lipid content and eADA activity suggest detrimental effect of BCFA supplementation.

**Supplementary Information:**

The online version contains supplementary material available at 10.1007/s11033-025-11220-9.

## Introduction

It is generally known that the composition of fatty acids (FA) in the diet influences the risk of atherosclerosis. Polyunsaturated fatty acids n-3 (n-3 PUFAs), such as eicosapentaenoic and docosahexaenoic acids (EPA and DHA), are particularly well studied and are associated with numerous health benefits, including anti-atherosclerosis and anti-inflammatory properties [1]. Much less is known about branched-chain fatty acids (BCFAs), which are characterized by additional methyl groups located at 2nd (iso-BCFA) or 3rd (anteiso BCFA) carbon atom at methyl end of hydrocarbon chain of BCFA. The major source of BCFA in human body is the diet, particularly the dairy products, ruminant milk or butter [2, 3]. The daily intake of BCFA is estimated 400–600 mg/day [4]. We can expect that BCFA intake in rodents is much lower, because of consumption of high-carbohydrate diet with much lower content of lipids. However, these FA can be also synthetized by intestinal bacteria [5] and by mammals’ cells [6]. Preliminary studies, mostly in human hepatocellular carcinoma cell line HepG2, suggest that iso-BCFAs may have numerous beneficial properties, including anti-inflammatory and lipid-lowering [7]. Human and animal studies revealed that blood levels of certain iso-BCFAs are inversely correlated with triglyceride concentrations and C-reactive protein (CRP) [8, 9]. Our previous study showed that 14-methylpentadecanoic acid, a representative of iso-BCFA decreased the expression of fatty acid synthase (FASN) and c-reactive protein (CRP) genes in HepG2 hepatocytes, that suggest that this BCFA can reduce lipogenesis and inflammation in liver [10]. However, to verify these findings in vivo, extensive studies in animal models are required. Genetically modified mice with silenced genes for apolipoprotein E and the LDL receptor – double knockout (*ApoE*^*−/−*^*/Ldlr*^*⁻/⁻*^) is one of the models suitable for the study of the effect of diet supplements on atherosclerosis development. These mice show hyperlipidaemia and spontaneous development of atherosclerosis without the need for a high-fat diet treatment [11]. It should be noted that *ApoE*^*−/−*^*/Ldlr*^*⁻/⁻*^mice have significantly increased TC, TG and LDL-C compared to normal mice [12]. The aim of this study was to evaluate if supplementation of (*ApoE*^*−/−*^*/Ldlr*^*⁻/⁻*^) mice with iso-BCFA in diet result in improvement of hyperlipidaemia and affect serum and tissue FA profile. Moreover, we studied the effect of BCFA supplementation in these mice on lipid content in aorta and ecto-adenosine deaminase (eADA) activity.

## Materials and methods

### Animal maintenance and housing

Male *ApoE*^*−/−*^*/Ldlr*^*⁻/⁻*^ mice aged nine (*n* = 20) months were randomly divided into two experimental groups (controls and iso-BCFA treated). The animals treated by iso-BCFA received diet enriched by 13-methyldecanoic acid (13-MTA; 0.800 g/kg diet), 14-methyldecanoic acid (14-MTA; 0.800 g/kg diet) and 15-methylhexanoic acid (15-MHA; 1.000 g/kg diet) and corn oil (67.4 g/kg diet), while control mice were fed standard chow (Zoolab, Poland). The dose of supplemented iso-BCFA was selected based on a preliminary study. The animals were kept in an alternating 12-hour cycle (light/dark) in individually ventilated cages (IVC) with an area of 432 cm^2^ (22.5 ± 0.5 °C, 55 ± 5% humidity) with the number of animals not exceeding 5 in one cage. The animals were provided with filtered supply and exhaust air and a constant airflow velocity of approximately 0.1 m/s. Mice had unlimited access to water and food. The weight of the mice and food intake were measured twice a week. During the experiment, blood was drawn from all mice at the second, fourth, eighth and twelfth experimental weeks. The blood was centrifuged at 4000 rpm for 10 min at 20 °C and the serum samples were aliquoted. After twelve weeks, the mice were sacrificed under anaesthesia by intraperitoneal injection of xylazine (70 mg/kg) and ketamine (6 mg/kg). The age of mice at the end of experiment was 12 months. Isolated fragments of the aorta were split off from the perivascular adventitia in cold HBSS, cut along the inner surface and used to measure eADA activity. The heart and liver were removed and immediately frozen in liquid nitrogen. All samples were stored at −80 °C until analysis.

### Serum biochemical parameters analysis

Total cholesterol (TC), low-density lipoprotein cholesterol (LDL-C), high-density lipoprotein cholesterol (HDL-C), triacylglycerol (TG) and high-sensitivity C-reactive protein (hs-CRP) were measured using an automated photometer (ERBA XL-180, Mannheim, Germany) and specific ERBA kits according to the manufacturer’s instructions.

### Fatty acid profile analysis

Total lipids were extracted from tissues, including liver, heart and abdominal aorta, and from serum using a chloroform: methanol mixture (2:1, v/v) according to the method of Folch et al. [13]. The chloroform phase was collected and evaporated under nitrogen. The extracted lipids were hydrolysed in 1 mL of 0.5 M KOH in methanol at 90 °C for 3 h. The reaction mixture was then acidified with 0.2 mL of 6 M HCl, followed by the addition of 1 mL of water. The FA were extracted three times with 1 mL n-hexane and dried under nitrogen. For derivatization, the hydrolysed FA were converted to fatty acid methyl ester (FAME) with a 10% boron trifluoride-methanol solution at 55 °C for 1.5 h. After the reaction, 1 mL of water was added and the FAMEs were extracted three times with 1 mL of n-hexane and dried under nitrogen. The FAMEs produced were analysed using a GC-EI-MS QP-2020NX (Shimadzu, Japan). Separation was performed on a Zebron ZB-5MSi capillary column (30 m × 0.25 mm i.d. × 0.25 μm film thickness). The temperature of the GC oven was programmed from 125 °C to 300 °C at a rate of 4 °C/min, with a total run time of 46.5 min. Helium served as the carrier gas with a column head pressure of 100 kPa. Mass spectrometry was performed with an electron impact ionization source at 70 eV. The mass spectra were recorded in full scan mode over an m/z range of 45–700. 19-methylarachidic acid was used as an internal standard, manual integration was used to identify FAs for each FA peak, and FAs were identified using FA reference standards (Larodan, MI, USA and Merck, Darmstadt, Germany).

### The measurement of the activity of ecto-adenosine deaminase (eADA) on the surface of the abdominal and thoracic aorta

The abdominal and thoracic aorta was isolated from *ApoE*^*−/−*^*/Ldlr*^*⁻/⁻*^ mice as previously described by Kutryb-Zajac et al. [14]. Vessels were rinsed three times with Hank’s Balanced Salt Solution (HBSS, Sigma Aldrich No. H6648) and pre-incubated in a 24-well plate with 1 mL HBSS at 37 °C. Adenosine was then added to each well at a final concentration of 50 µM. To examine eADA, 50 µL supernatant was taken after 0, 5, 15, and 30 min. The samples obtained were immediately analysed by reversed-phase high-performance liquid chromatography (RP-HPLC). The reaction velocity was calculated based on the linear range and normalized to tissue weight.

### Quantification of atherosclerotic lesions

Atherosclerotic lesions were indirectly quantified in the thoracic aorta of *ApoE*^*−/−*^*/Ldlr*^*⁻/⁻*^ mice by staining with the neutral lipid-targeting lysochrome Oil red O (ORO), as previously described by Beetie et al. [15]. The aorta of mice was rinsed in distilled water and 70% isopropanol, followed by 30 min of ORO staining. Subsequently, the stained aortas were rinsed in 70% 2-propanol and transferred to distilled water. Stained aortas were placed in 2 ml glass vials containing 200 µl chloroform/methanol mixture (2:1, v/v). The vials were placed on the shaker for 10 min. When all the staining had dissolved, the buffer was transferred to a 96-well polypropylene plate and ORO was measured at 490 nm using a microplate reader (Biotek, Santa Clara, USA). The amount of ORO in µM released from the surface of the aortas was calculated using a standard curve of ORO (1, 10, 50, 100 µM) prepared in chloroform/methanol.

### Analysis of mRNA levels

Total RNA was extracted from frozen liver or heart using the RNeasy Plus Universal Mini Kit (Qiagen, Netherlands) according to the attached protocol. After the final centrifugation, the RNA was washed with 40 µl elution solution. A NanoDropOne spectrophotometer (ThermoFisher Scientific, Waltham, MA, USA) was used to analyse RNA concentration and purity. RNA was reversed using random hexamer primers (RevertAid First Strand cDNA Synthesis Kit, Thermo Fisher Scientific, Waltham, MA, USA) according to the manufacturer’s instructions. Duplicates of each sample were analysed by real-time polymerase chain reaction (RT-PCR) using a CFX Connect Real-Time System (Bio-Rad, Hercules, CA, USA). β-actin was used as the reference gene. At the end of the amplification cycle, a melting analysis was performed to check for nonspecific amplification. The cycle number at which a particular sample exceeded this threshold (Ct) was then used to determine the fold difference. The fold difference was calculated as 2–ΔΔCt. The sequences for the RT-PCR primers were as follows (5’−3’): β-actin RT-F: CCAGTTTGGTAACAATGCCATGT; β-actin RT-R: GGCTGTATTCCCCTCCATCG; BCKDHA RT-F: AGTCCCTGCCCTTGTACACA; BCKDHA RT-R: CGATCCGAGGGCCTCACTA; BCKDHB RT-F: GTTGCCTTTGGTGGAGTCTTCC; BCKDHB RT-R: GTGACCGCGATTCCAATGCCAA; BCAT 2 RT-F: CAAAGGTGGAGACCAGCAGGTA; BCAT 2 RT-R: TGGCGGATACACTCCAACAGCT; CRP RT-F: GGCCAGATGCAAGCATCATC; CRP RT-R: CTGGAGATAGCACAAAGTCCCAC; CPT1 RT-F CGTGCTGCTTTCTTTGTG; CPT1 RT-R AGTGTTCGGTGTTGAGGC; ACC RT-F CCGTTGGCCAAAACTCTGGAGCTAA; ACC RT-R GAGCTGACGGAGGCTGGTGACA; FASN RT-F CTCATTGGCCTGGGCGG; FASN RT-R ACCTCCTCCATGGCTCTTCT; IL-6 RT-F TCTGCAAGAGACTTCCATCCA; IL-6 RT-R CAGGTCTGTTGGGAGTGGTA; LPL RT-F GCGTAGCAGGAAGTCTGACCAA; LPL RT-R AGCGTCATCAGGAGAAAGGCGA; CD36 RT-F GGACATTGAGATTCTTTTCCTCTG; CD36 RT-R CAAAGGCATTGGCTGGAAGAAC; FATP1 RT-F TGCCACAGATCGGCGAGTTCTA; FATP1 RT-R AGTGGCTCCATCGTGTCCTCAT; PPARa RT-F TGCCTTCCCTGTGAACTGAC; PPARa RT-R TGGGGAGAGAGGACAGATGG, EHHADH RT-F TGGACCATACGGTTAGAGCC; EHHADH RT-R GATCCTGCGGGGTTCTATGG; VNN1 RT-F: GTCCTTCCTCATTTGGCAGCCA; VNN1 RT-R: CTGTCGTAGTGAAAGACCCTTGG; HMGCS2 RT-F TGCTATGCAGCCTACCGCAAGA; HMGCS2 RT-R GCCAGGGATTTCTGGACCATCT; CYP4A10 RT-F GCTACTCAAGGCTTTCCAGCAG; CYP4A10 RT-R CCAGAACCATCTAGGAAAGGCAC; FGF21 RT-F ATCAGGGAGGATGGAACAGTGG; FGF21 RT-R AGCTCCATCTGGCTGTTGGCAA.

### Statistical analysis

Differences in serum iso-BCFA concentrations in mice supplemented via diet during the experiment were calculated using an ANOVA on ranks test for multiple comparisons, followed by Dunn’s post hoc analysis. Other calculations regarding differences in neutral lipid content, vascular adenosine deamination activity, body, liver, and adipose tissue mass, as well as serum biochemical parameters and mRNA expression levels, were performed using two-sample t-tests, followed by a Mann-Whitney rank-sum test. Differences were considered significant at *p* < 0.05. Data are presented as median (Q1-Q3).

## Results

###  The effect of BCFA supplementation on serum and tissue BCFA concentrations

**Fig. 1:**
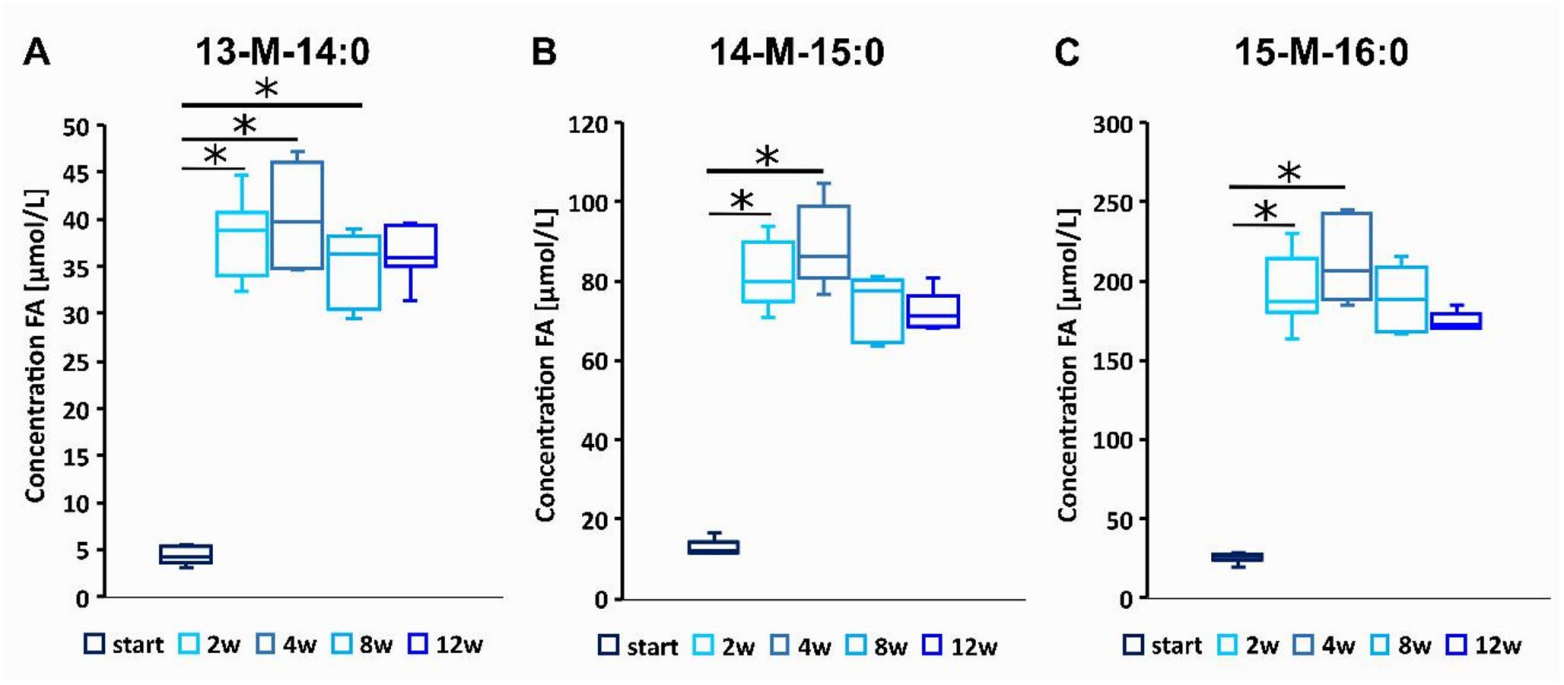
Serum concentrations of iso-BCFA (µmol/L) supplemented in diet during experiment: (**A**) 13-methyl-14:0, (**B**) 14-methyl-15:0, and (**C**) 15-methyl-16:0 (*n* = 8). Statistical differences were calculated using the ANOVA on ranks test for multiple comparison followed by a Dunn’s post-hoc analysis and considering *p* < 0.05 significant, w – following weeks of the experiment, *p < 0.05

The analysis of the levels of iso-BCFA, that were supplemented in diet showed that after just 2 weeks their levels were strongly increased in blood of *ApoE*^*−/−*^*/Ldlr*^*⁻/⁻*^ mice and remained more or less stable to the end of experiment (Fig. 1). Increased levels of those BCFA were also found in liver, abdominal aorta and heart of BCFA supplemented mice at the end of experiment (Tables S1-S3). We have also found some minor differences in the levels of other FA than those supplemented in liver, abdominal aorta, heart and serum between *ApoE*^*−/−*^*/Ldlr*^*⁻/⁻*^ mice supplemented by iso-BCFA and those fed by standard diet (Tables S1-S4).

**Table 1:** Body, liver and adipose tissue mass and serum biochemical parameters in BCFA-fed and SD-fed ApoE^−/−^/LDLR^-/-^ mice at start and end of the experiment

Parameter	SD-fed start	SD-fed end	BCFA-fed start	BCFA-fed end	SD-fed start vs. end	BCFA-fed start vs. end	SD-fed end vs. BCFA-fed end
Weight [g]	31.6 (29.6–32.0)	31.3 (29.7–31.7)	30.8 (28.9–34.4)	29.6 (29.0–32.6.0.6)	ns	ns	ns
Liver mass [g]	-	1.44 (1.28–1.56)	-	1.45 (1.14–1.65)	-	-	ns
Adipose tissue mass [g]	-	1.17 (0.83–1.59)	-	0.97 (0.5–1.45)	-	-	ns
Liver lipid content [mg/g]	-	38.7 (25.6–48.1)	-	41.2 (31.6–54.8)	-	-	ns
LDL-C [mg/dL]	347 (321–410)	531 (371–561)	429 (382–454)	349 (324–404)	ns	ns	ns
HDL-C [mg/dL]	28.7 (21.7–30.1)	42.0 (38.5–48.3)	32.2 (31.0–39.7.0.7)	34.3 (30.4–41.3)	0.030	ns	ns
Remnant C	561 (519–642)	510 (436–551)	458 (379–529)	336 (320–404)	ns	0.020	0.017
TC [mg/dL]	833 (812–889)	1092 (872–1130)	913 (800–1013)	724 (690–816)	ns	0.020	0.030
TG [mg/dL]	253 (190–313)	424 (401–449)	323 (281–382)	225 (169–283)	0.005	0.008	0.004
hs-CRP [mg/L]	1.12 (0.84–1.33)	1.12 (0.94–1.30)	1.33 (1.05–1.40)	1.19 (0.94–1.44)	ns	ns	ns
TG/HDL-C ratio	8.42 (5.80–14.2)	9.77 (8.50–11.7)	10.3 (8.37–12.1)	6.12 (4.06–8.47)	ns	0.013	0.030
LDL-C/HDL-C ratio	12.1 (7.96–18.4)	10.4 (9.39–13.2)	13.7 (11.1–14.0)	10.2 (9.39–11.4)	ns	0.005	ns
TC/HDL-C ratio	29.0 (20.2–37.3)	25.2 (20.2–26.5)	27.6 (24.1–31.4)	21.2 (19.8–23.4)	ns	0.020	ns

The results are presented as median (Q1-Q3); SD-fed start n = 7, SD-fed end n = 5, BCFA-fed start and BCFA-fed end n = 8; HDL-C – high density lipoprotein cholesterol, hs-CRP – high-sensitivity C-reactive protein, LDL-C – low density lipoprotein cholesterol, Remnant C – remnant cholesterol (total cholesterol minus HDL-C and LDL-C), TC – total cholesterol, TG – triacylglycerol, ns – no significant

### Impact of iso-BCFA on body weight and serum biochemical parameters

The body mass did not change significantly during experiment both in SD and BCFA fed *ApoE*^*−/−*^*/Ldlr*^*⁻/⁻*^ mice. The body mass changes measured week by week are presented in Figure S1. As it was mentioned, *ApoE*^*−/−*^*/Ldlr*^*⁻/⁻*^mice exhibit markedly elevated concentrations of TC, TG, and LDL-C. BCFA treatment caused slight decrease of serum LDL-C (non-significant) and a significant decrease of serum TG, remnant cholesterol and TC at the end point compared to start point (Table 1). At the same time, in control mice HDL-C, LDL-C and total cholesterol (non-significantly) increased during the experiment, that was probably associated with atherogenic changes development (Table 1). Importantly, parameters associated with atherosclerosis development such as LDL-C/HDL-C, TC/HDL-C and TG/HDL-C also improved in the BCFA supplementation group (Table 1).

### BCFA supplementation increases eADA activity and lipid content in vascular tissue

**Fig. 2:**
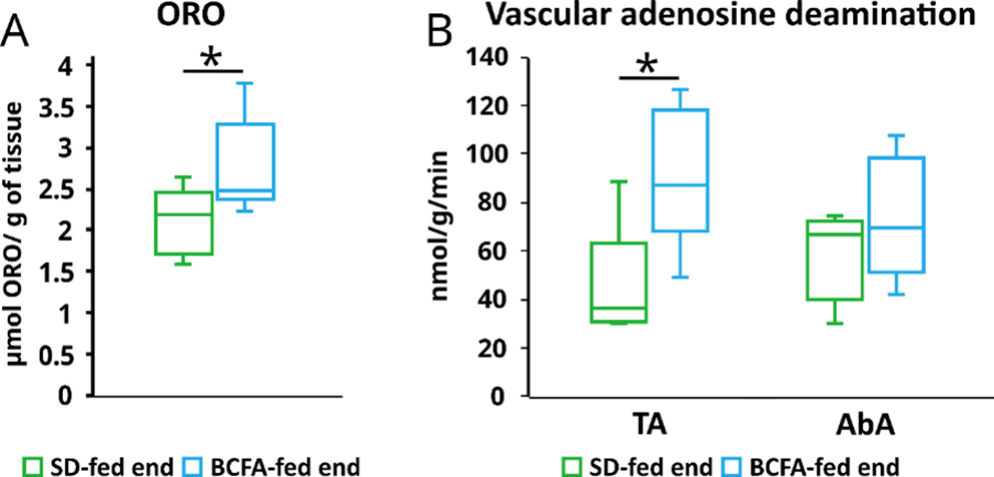
Neutral lipid content assessment by ORO staining (**A**) and vascular adenosine deamination (eADA activity) (**B**) in aorta of *ApoE*^*−/−*^*/Ldlr*^*⁻/⁻*^ mice fed standard diet (SD) and iso-BCFA enriched diet (BCFA). *p < 0.05 from nonparametric Mann-Whitney test on rank, TA - thoracic aorta, AbA - abdominal aorta

Then, a study of lipid content and eADA activity revealed that both parameters increased in thoracic aorta (TA) (Fig. 2). Moreover, eADA activity tended to increase in abdominal aorta (AbA) (Fig. 2).

### The effects of BCFA supplementation on the expression of selected genes involved in lipid metabolism and inflammation in liver

**Fig. 3:**
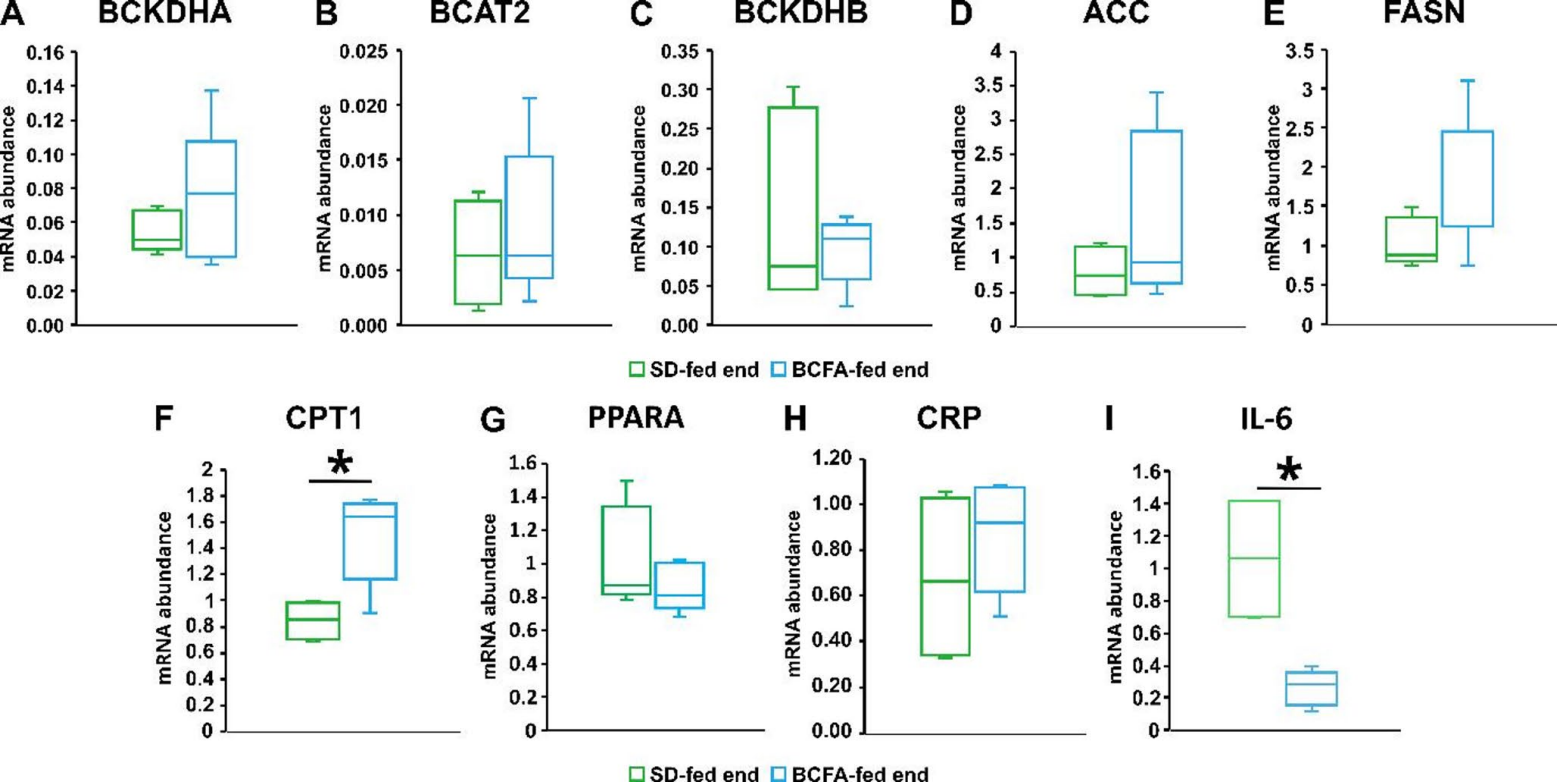
mRNA relative levels of selected genes involved in lipid metabolism and inflammation in the livers from BCFA-fed and SD-fed *ApoE*^*−/−*^*/Ldlr*^*⁻/⁻*^ mice: (**A**) *Bckdha*, (**B**) *Bcat2*, (**C**) *Bckdhb*, (**D**) *Acc*, (**E**) *Fasn*, (**F**) *Cpt1*,(**G**) *PPARa*, (**H**) *CRP*, (**I**) *Il-6*. *p < 0.05, p value from two-sample t-tests, followed by Mann-Whitney rank-sum test

To verify the effect of iso-BCFA on lipid metabolism and inflammation we have also measured the expression of genes associated with both conditions in the livers of *ApoE*^*−/−*^*/Ldlr*^*⁻/⁻*^ mice. Liver is a central organ in lipid metabolism and a site of CRP production. We did not find any effect of iso-BCFA supplementation on *CRP* gene expression (Fig. 2), which is consistent with the lack of effects on serum concentration of this protein. However, the level of mRNA of another cytokine associated with inflammation – *Il6* was significantly lower in mice supplemented by iso-BCFA (Fig. 2H). We did not find any effect of iso-BCFA supplementation on the mRNA levels of genes encoding enzymes of branched chain amino acid (BCAA) catabolism – branched chain ketoacid dehydrogenase A and B (Bckdha, Bckdhb) and branched chain amino acid aminotransferase 2 (Bcat2). Products of reactions catalysed by these enzymes may serve as a substrate for BCFA synthesis. The mRNA levels of lipogenic enzymes – fatty acid synthase (Fasn) and acetyl-CoA carboxylase (Acc) tended to increase after iso-BCFA supplementation, but these trends were nonsignificant. The most pronounced and statistically significant was the elevation of mRNA level of carnitine-palmitoyltransferase 1 (*Cpt1*), an enzyme critical for the regulation of FA β-oxidation. Also, the upstream gene *PPARa* that potentially may regulate the expression of *Cpt1* was measured in liver, however, we have found that BCFA treatment did not change the mRNA level of *PPARa* significantly (Fig. 3). Moreover, the mRNA levels of downstream genes regulated by PPARa (including *Ehhadh*,* Vnn1*,* Hmgcs2*,* Cyp4a10*, and *Fgf21*) has been analysed, but we did not find any significant differences between mice treated or non-treated by iso-BCFA (Figure S2).

**Fig. 4:**
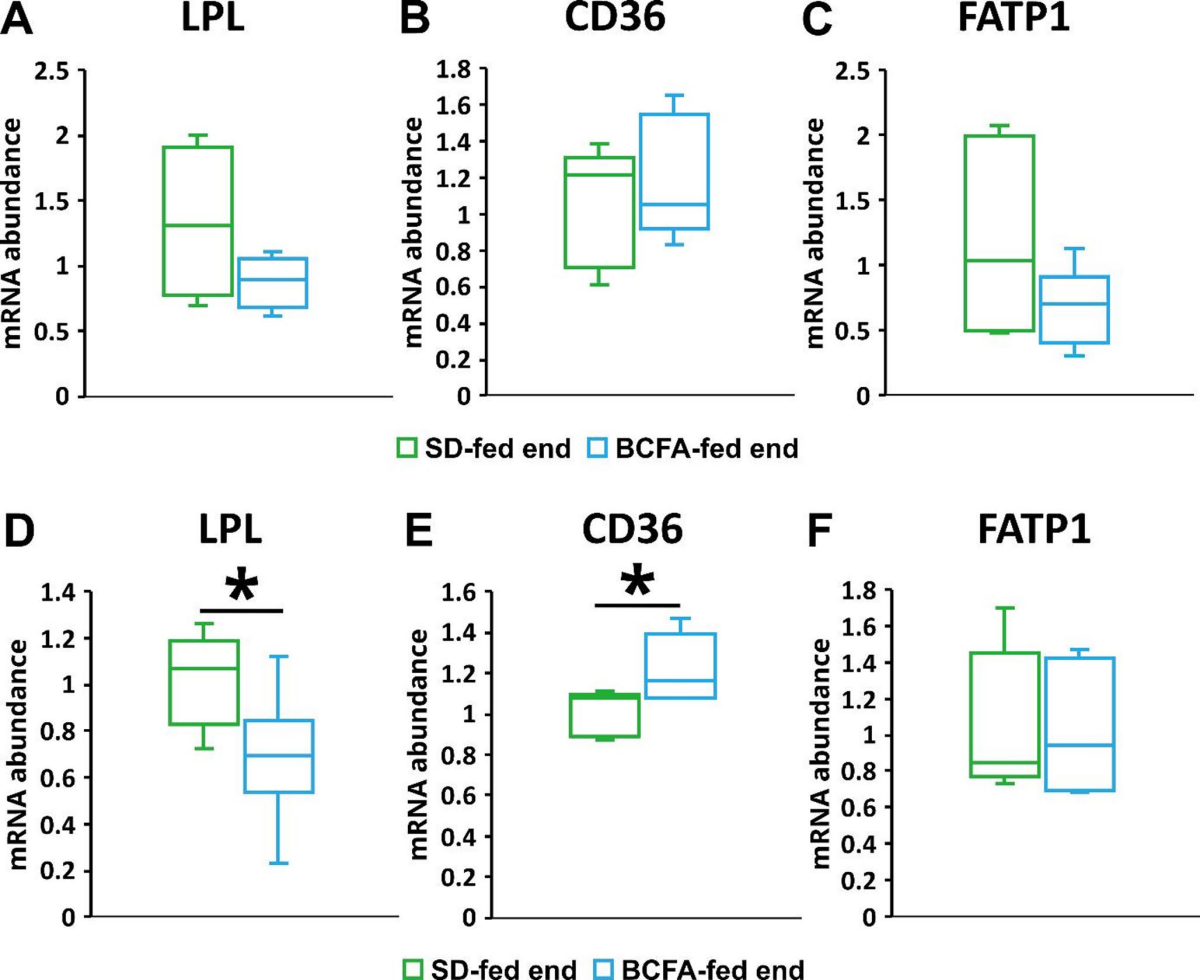
mRNA relative levels of selected genes involved in lipolysis of TG-rich proteins and FA transporters in the skeletal muscles (**A**) *Lpl*, (**B**) *Cd36*, (**C**) *Fatp1*, and adipose tissue (**D**) *Lpl*, (**E**) *Cd36* (**E**) *Fatp1* from BCFA-fed and SD-fed *ApoE*^*−/−*^*/Ldlr*^*⁻/⁻*^ mice. *p < 0.05, p value from two-sample t-tests, followed by Mann-Whitney rank-sum test

To verify if changes in lipolysis of TG -rich proteins may contribute do the decrease of serum TG after BCFA treatment we have measured the expression of lipoprotein lipase (LPL) and FA transporters (CD36 and FATP1) in muscle and adipose tissue, that are the major tissues up taking FA from TG-rich lipoproteins. We have found decreased mRNA levels of Lpl both in muscle (non-significantly) and adipose tissue after BCFA treatment, whereas CD36 did not change in muscle and increased in adipose tissue, and Fatp1 did not change significantly in both tissues (Fig. 4).

## Discussion

This study showed for the first time the effects of iso-BCFA supplementation on metabolic parameters in atherosclerotic *ApoE*^*−/−*^*/Ldlr*^*⁻/⁻*^ mice. We have chosen this model of atherosclerosis due to hyperlipidaemia and inflammation [12, 16]. The premise to use iso-BCFA were our previous in vitro experiments, in which we found that iso-BCFA can decrease the expression of FASN and CRP genes in hepatocytes [10]. First of all, we showed that dietary iso-BCFA supplementation was effective - we found a significant increase in BCFA in the blood (approximately 4-fold) and in tissues (liver, heart and aorta). The increased levels of iso-BCFA in blood were stable throughout the whole treatment. In serum of healthy human, the total BCFA concentration is about 15 mg/L [17], which corresponds to approximately 60 µmol/L. The concentration of total BCFA in mice supplemented by iso-BCFA is about 120 µmol/L. Consistently, the daily intake of BCFA in human is estimated 400–600 mg/day [4] (corresponding to approximately 0.2% of total energy intake) whereas in mice supplemented by iso-BCFA it is about 10 mg/day, (corresponding to about 0.6% of total energy intake). These data suggest that potential supplementation of BCFA in human may result in similar effect on the BCFA concentration in serum like in mice, since for example using commercially available omega-3 FA supplements (EPA + DHA) in human led to 2–3 time increase of their levels in blood [18].

The most important result of this study was the reduction of hyperlipidaemia after 12 weeks of the BCFA-enriched diet in *ApoE*^*−/−*^*/Ldlr*^*⁻/⁻*^mice. Particularly spectacular was reduction of TG levels in the blood by about 30% in iso-BCFA treated mice, whereas at the same time the TG concentrations in SD-mice increased by 70%. Also, the levels of total cholesterol were decreased in iso-BCFA fed mice, and the levels of LDL-C tended to decrease, whereas in SD mice it increased significantly during the experiment. The above changes in serum lipids of mice supplemented by BCFA may be antiatherogenic and cardioprotective. Our results are consistent with previous studies performed on mice [19] and C. elegans [20], which showed that the TG content in the liver and other organs decreased as a result of the increase in BCFA content. To study the mechanism of decreased TG levels in blood we have analyzed the expression of genes related to lipogenesis (*Fasn* and *Acc*) and FA β-oxidation (*Cpt1*) in the liver of iso-BCFA treated and SD mice. In contrast to our previous studies on HepG2 hepatocytes [10] we did not find a decrease in the expression of lipogenic enzymes. This may be due to the fact that liver cells of mice, which were used in the current experiment, may differentially response to iso-BCFA treatment than HepG2 hepatocytes, possibly due to knock-out of *ApoE* and *Ldlr* genes. However, the increased expression of *Cpt1* in liver of BCFA treated *ApoE*^*−/−*^*/Ldlr*^*⁻/⁻*^mice may be a possible explanation of lowering of serum lipids in this group. The utilization of FA in mitochondria may decrease their content in liver, and thus, decrease liver VLDL production and export, that could result in lower levels of blood TG and cholesterol. The expression of upstream regulator of *Cpt1* - PPARa did not changed after BCFA enriched diet. Since PPARa activity may be regulated post-transcriptionally, we have also measured the mRNA levels of its downstream genes including *Ehhadh*,* Vnn1*,* Hmgcs2*,* Cyp4a10*, and *Fgf21*, but we did not find any significant differences between mice treated and non-treated by iso-BCFA. Together, these data suggest that other regulation may be responsible for *Cpt1* overexpression. Despite the fact that some mechanism of BCFA action on various cells have been proposed, this issue is very little studied. Generally, it is believed that they might influence various signalling pathways, like those with mTOR1 and SREBP1c [7]. It also cannot be excluded that they may act indirectly, and their effects may be related to some metabolites produced from BCFA like propionyl-CoA or branched chain acylcarnitines. Another reason of the decrease of blood TG after iso-BCFA rich diet might be the increased TG uptake from TG-rich lipoproteins to tissues. However, the results of the measurement of LPL and FA transporting proteins suggest that the decrease of serum TG after BCFA treatment is not related to increased uptake of TG from TG-rich lipoproteins. This is consistent with a trend to decrease of adipose tissue mass in BCFA treated mice.

Our expectation was that decreased blood lipids after high-iso-BCFA diet would result in improvement of aorta condition. Unexpectedly, we found higher levels of lipids in aorta of iso-BCFA treated mice and higher adenosine catabolism, that reflects vascular adenosine deaminase (eADA) activity. The eADA activity is increased in *ApoE*^*−/−*^*/Ldlr*^*⁻/⁻*^ mice when compared with wild type and contributes to atherosclerosis progression [14]. These two parameters, unfortunately, suggest that iso-BCFA treatment, despite improvement of blood lipid profile worsens the aorta condition. Increased lipid content in aorta may contribute to the development of atherosclerotic plaque, and reduced adenosine levels may result in endothelial dysfunction. Thus, positive effect of iso-BCFA supplementation on hyperlipidaemia in atherosclerotic mice co-occurs with negative effects in aorta.

Another parameter that we expected to be modified by iso-BCFA was CRP – a marker of inflammation. In HepG2 cells the treatment by iso-BCFA caused decrease of *CRP* mRNA levels [10]. However, in BCFA treated mice we did not find any significant changes in serum CRP concentrations nor *Crp* gene expression in liver, which is a major source of this protein. Again, it may be due to different response to iso-BCFA treatment in HepG2 hepatocytes, and liver of mice with of ApoE and LDLR genes knock-out. Some authors suggest that CRP is not a good marker of inflammation in mice, and that *Il6* is better in this respect [21]. Our results show much lower mRNA level of *Il6* that suggest decreased inflammation in liver. It is consistent with our previous study in iso-BCFA treatment of human hepatocytes [10]. Unfortunately, our biochemical analyser ERBA does not allow to measure *Il6*, and we had no more serum to perform ELISA measurement of *Il6*. Thus, our results are limited to *Il6* gene expression in liver and we can only speculate that it may also decrease serum *Il6* levels.

Our study also showed that iso-BCFA treatment caused mild alterations of FA profiles in serum and studied tissue. These changes were not limited to BCFA, that was expected but also they were significant in case of some representative of other groups of FA, like very long chain saturated and monounsaturated FA, odd chain FA or PUFA. This suggest that iso-BCFA can have some pleiotropic effects, that remains to be studied.

This study has some limitations. The study was carried out on male mice, which are characterized by lower inter-individual variability; however, this does not provide an insight BCFA supplementation in females. Additionally, animals were not starved before blood collection that may influence the concentrations of lipids, however the content of lipids in chow in both groups was low (0.26%), and thus, it should not affect serum lipids significantly. Another limitation of our study is that atherosclerosis was assessed indirectly by Oil Red O staining of aortic sections, which reflects vascular lipid deposition but does not provide a direct quantitative measure of plaque burden. Therefore, our findings should be interpreted in terms of relative differences in vascular lipid accumulation between groups rather than atherosclerosis plaque burden. Moreover, the inflammatory markers in the aortas of mice were not analysed due to the small amounts of tissue available.

## Conclusions

Our study did not provide a clear answer as to whether iso-BCFA treatment can be effective in preventing atherosclerosis in a mouse *ApoE*^*−/−*^*/Ldlr*^*⁻/⁻*^model. While improving blood lipids, at the same time it caused increased lipid content and eADA activity in aorta of mice. Further in-vivo studies are needed to elucidate if iso-BCFA supplementation have a potential to prevent atherosclerosis, possibly in other animal models of atherosclerosis.

## Supplementary Information

Below is the link to the electronic supplementary material.

Supplementary Material 1

## Data Availability

The datasets used and analyzed during this study are available at [https://repozytorium.bg.ug.edu.pl/info/researchdata/UOG79a81bc0aa98432c98d2b1f7c2d1abd7/](https:/repozytorium.bg.ug.edu.pl/info/researchdata/UOG79a81bc0aa98432c98d2b1f7c2d1abd7).
