# Supplementary material for: The impact of supplementation with iso-branched chain fatty acids in a mouse model of atherosclerosis
Source: Mol Biol Rep. 2025 Nov 12;53(1):71. doi: 10.1007/s11033-025-11220-9 (PMC12611978; doi:10.1007/s11033-025-11220-9)
Supplement: Supplementary file 1 — Supplementary Material 1 [file 11033_2025_11220_MOESM1_ESM.docx]

**Does the supplementation of *iso-*branched chain fatty acids protect from cardiovascular risk in mouse model of atherosclerosis ?**

Agata Zwara^a^, Agata Jedrzejewska^b^, Dorota Olszewska^c^, Marta Tomczyk^b^, Tomasz Sledzinski^c^, *****Adriana Mika^a,c^

a Department of Environmental Analytics, Faculty of Chemistry, University of Gdansk, Wita Stwosza 63, 80-308 Gdansk, Poland

b Department of Biochemistry, Faculty of Medicine, Medical University of Gdansk, Debinki 1, 80-211 Gdansk, Poland

c Department of Pharmaceutical Biochemistry, Faculty of Pharmacy, Medical University of Gdansk, Debinki 1, 80-211 Gdansk, Poland

***Corresponding author:** adriana.mika@gumed.edu.pl

Table S1: Content (% of total FA) of FA in the liver of 12-month-old SD-fed and BCFA-fed mice

| **FA** | **SD-fed mice** | **BCFA-fed mice** | **p value** |
| --- | --- | --- | --- |
| 10:0 | traces | traces | - |
| 12:0 | 0.020 (0.020; 0.025) | 0.030 (0.023; 0.040) | 0.065 |
| 14:0 | 0.46 (0.43; 0.66) | 0.59 (0.37; 0.75) | 0.622 |
| 16:0 | 21.8 (20.9; 22.1) | 21.4 (19.9; 21.7) | 0.354 |
| 18:0 | 11.1 (9.02; 11.7) | 10.1 (8.85; 13.1) | 0.943 |
| 20:0 | 0.65 (0.46; 0.82) | 0.50 (0.39; 0.60) | 0.284 |
| 22:0 | 0.31 (0.25; 0.48) | 0.28 (0.19; 0.34) | 0.354 |
| 24:0 | 0.32 (0.22; 0.41) | 0.26 (0.18; 0.34) | 0.435 |
| 26:0 | 0.015 (0.010; 0.028) | 0.020 (0.010; 0.020) | 1.000 |
| **Total ECFA** | **34.5 (31.9; 35.7)** | **32.7 (32.0; 36.2)** | **0.833** |
| 11:0 | traces | traces | - |
| 13:0 | traces | traces | - |
| 15:0 | 0.16 (0.13; 0.20) | 0.17 (0.16; 0.22) | 0.435 |
| 17:0 | 0.26 (0.26; 0.38) | 0.32 (0.27; 0.40) | 0.435 |
| 19:0 | 0.17 (0.12; 0.20) | 0.16 (0.12; 0.22) | 0.943 |
| 21:0 | 0.020 (0.010; 0.025) | 0.020 (0.020; 0.030) | 0.354 |
| 23:0 | 0.080 (0.050; 0.085) | 0.060 (0.042; 0.090) | 0.943 |
| 25:0 | 0.025 (0.012; 0.030) | 0.020 (0.010; 0.025) | 0.556 |
| **Total OCFA** | **0.66 (0.62; 0.83)** | **0.81 (0.68; 1.01)** | **0.109** |
| 2,5,10-triM-12:0 | 0.010 (0.010; 0.010) | 0.010 (0.010; 0.020) | 0.429 |
| 4,8,12-triM-14:0 | 0.010 (0.010; 0.010) | 0.010 (0.010; 0.010) | 0.788 |
| **Total triM BCFA** | **0.020 (0.015; 0.020)** | **0.020 (0.020; 0.030)** | **0.432** |
| 12-M-13:0 | 0.010 (0.010; 0.010) | 0.010 (0.010; 0.010) | 1.000 |
| 13-M-14:0 | 0.020 (0.020; 0.020) | 0.090 (0.080; 0.14) | **0.002** |
| 14-M-15:0 | 0.070 (0.055; 0.090) | 0.25 (0.23; 0.29) | **0.002** |
| 15-M-16:0 | 0.11 (0.10; 0.13) | 0.65 (0.51; 0.71) | **0.002** |
| 16-M-17:0 | traces | Traces | - |
| 17-M-18:0 | 0.050 (0.040; 0.050) | 0.18 (0.12; 0.22) | **0.002** |
| 18-M-19:0 | 0.030 (0.025; 0.040) | 0.045 (0.032; 0.058) | 0.093 |
| 20-M-21:0 | 0.010 (0.010; 0.015) | 0.010 (0.010; 0.010) | 0.943 |
| 22-M-23:0 | 0.015 (0.010; 0.020) | 0.025 (0.012; 0.038) | 0.214 |
| **Total iso BCFA** | **0.32 (0.28; 0.35)** | **1.29 (1.06; 1.46)** | **0.002** |
| 10-M-12:0 | traces | traces | - |
| 12-M-14:0 | 0.020 (0.015; 0.030) | 0.020 (0.020; 0.030) | 0.931 |
| 14-M-16:0 | 0.020 (0.015; 0.030) | 0.030 (0.020; 0.030) | 0.435 |
| 16-M-18:0 | 0.040 (0.030; 0.060) | 0.060 (0.058; 0.062) | 0.126 |
| 18-M-20:0 | traces | traces | - |
| 20-M-22:0 | 0.015 (0.010; 0.028) | 0.035 (0.020; 0.040) | 0.109 |
| 22-M-24:0 | traces | 0.010 (0.010; 0.018) | - |
| **Total anteisto BCFA** | **0.090 (0.080; 0.14)** | **0.14 (0.092; 0.17)** | **0.284** |
| **Total BCFA** | **0.43 (0.39; 0.50)** | **1.44 (1.22; 1.62)** | **0.002** |
| **Total SFA** | **35.7 (32.9; 37.0)** | **34.8 (34.0; 38.7)** | **0.833** |
| 14:1 | 0.030 (0.020; 0.030) | 0.030 (0.020; 0.040) | 0.876 |
| 16:1 | 2.71 (2.44; 3.66) | 3.58 (3.14; 4.43) | 0.126 |
| 17:1 | 0.040 (0.030; 0.070) | 0.060 (0.060; 0.092) | 0.222 |
| 18:1 | 18.3 (15.9; 21.6) | 19.9 (16.0; 22.8) | 0.833 |
| 19:1 | 0.010 (0.010; 0.020) | 0.010 (0.010; 0.020) | 1.000 |
| 20:1 | 0.94 (0.84; 0.96) | 0.84 (0.73; 1.04) | 0.435 |
| 22:1 | 0.070 (0.045; 0.10) | 0.060 (0.050; 0.080) | 0.724 |
| 24:1 | 0.20 (0.17; 0.30) | 0.14 (0.13; 0.18) | **0.030** |
| **Total MUFA** | **22.9 (19.8; 26.1)** | **24.3 (19.2; 28.3)** | **0.724** |
| HDA (16:2 n-6) | 0.050 (0.025; 0.060) | 0.030 (0.020; 0.040) | 0.202 |
| LA (18:2 n-6) | 23.5 (21.7; 25.2) | 20.5 (18.6; 22.9) | 0.127 |
| ARA (20:4 n-6) | 11.4 (8.89; 12.3) | 10.6 (9.78; 12.7) | 0.833 |
| DGLA (20:3 n-6) | 1.35 (1.22; 1.49) | 1.36 (1.15; 1.50) | 1.000 |
| EDA (20:2 n-6) | 0.50 (0.44; 0.66) | 0.44 (0.35; 0.50) | 0.171 |
| DPA n-6 (22:5 n-6) | 1.61 (1.28; 1.80) | 1.66 (1.26; 2.06) | 0.622 |
| AdA (22:4 n-6) | 0.99 (0.88; 1.08) | 0.83 (0.77; 0.99) | 0.093 |
| **Total PUFA n-6** | **38.5 (37.7; 39.8)** | **36.6 (34.1; 39.5)** | **0.284** |
| ALA (18:3 n-3) | 0.56 (0.44; 0.77) | 0.54 (0.33; 0.70) | 0.833 |
| EPA (20:5 n-3) | 0.14 (0.090; 0.14) | 0.14 (0.12; 0.16) | 0.354 |
| ETA (20:4 n-3) | 0.040 (0.025; 0.045) | 0.035 (0.030; 0.048) | 0.943 |
| DHA (22:6 n-3) | 2.33 (2.04; 2.58) | 2.58 (2.24; 3.04) | 0.354 |
| DPA n-3 (22:5 n-3) | 0.18 (0.16; 0.22) | 0.15 (0.14; 0.18) | 0.127 |
| **Total PUFA n-3** | **3.36 (2.96; 3.51)** | **3.46 (2.98; 3.97)** | **0.435** |
| **Supplemented iso-BCFA** | 0.20 (0.18; 0.24) | 1.06 (0.82; 1.09) | **0.002** |
| **Others iso-BCFA** | 0.11 (0.095; 0.12) | 0.29 (0.20; 0.36) | **0.002** |
| **C18:1/C18:0 ratio (SCD1)** | 1.65 (1.37; 2.40) | 1.97 (1.19; 2.61) | 0.833 |
| **ARA/DGLA ratio (D5D)** | 0.12 (0.12; 0.14) | 0.12 (0.11; 0.14) | 0.724 |
| **AdA/ARA ratio** | 0.094 (0.083; 0.10) | 0.080 (0.061; 0.097) | 0.222 |
| **ALA/LA ratio (D6D)** | 0.025 (0.019; 0.031) | 0.028 (0.017; 0.031) | 0.622 |
| **C22:0/C20:0 ratio (Elovl1)** | 0.56 (0.48; 0.60) | 0.52 (0.44; 0.60) | 0.524 |
| **C18:0/C16:0 ratio (Elovl6)** | 0.51 (0.42; 0.54) | 0.49 (0.42; 0.60) | 0.833 |
| **DHA/DPA n-3 ratio** | 12.1 (10.9; 13.5) | 16.3 (14.1; 18.3) | **0.011** |
| **ARA/LA ratio (Elovl5)** | 0.48 (0.36; 0.57) | 0.51 (0.46; 0.66) | 0.435 |

Value mediana (Q1; Q3); p value from non-parametric Mann-Whitney test on rank; AdA – adrenic acid, ALA - alpha-linolenic acid, ARA – arachidonic acid, BCFA – branched chain fatty acids, D5D - delta-5 desaturase; D6D - delta-6 desaturase  DGLA – dihomo-gamma-linolenic acid, DHA – docosahexaenoic acid, DPA – docosapentaenoic acid, ECFA – even chain fatty acids, EDA – eicosadienoic acid, ELOVL – elongase of fatty acid, EPA – eicosapentaenoic acid, ETA – eicosapentatetranoic acid, HDA – hexadecadienoic acid, LA – linoleic fatty acid, MUFA – monounsaturated fatty acids, OCFA – odd chain fatty acids, PUFA – polyunsaturated fatty acids, SCD1 - stearoyl-CoA desaturase 1; SFA – saturated fatty acids. Bold represents main groups of fatty acids.

Table S2: Content (% of total FA) of FA in the abdominal aortas of 12-month-old SD-fed and BCFA-fed mice

| **FA** | **SD-fed mice** | **BCFA-fed mice** | **p value** |
| --- | --- | --- | --- |
| 14:0 | 2.05 (1.40; 2.73) | 1.69 (1.57; 1.82) | 0.435 |
| 16:0 | 24.8 (23.2; 25.7) | 23.4 (22.4; 23.8) | 0.065 |
| 18:0 | 11.2 (10.9; 12.0) | 10.7 (9.78; 11.1) | 0.065 |
| 20:0 | 0.65 (0.62; 0.84) | 0.55 (0.51; 0.64) | 0.065 |
| 22:0 | 0.71 (0.58; 1.00) | 0.57 (0.44; 0.59) | **0.030** |
| 24:0 | 0.98 (0.84; 1.28) | 0.80 (0.60; 0.86) | 0.065 |
| 26:0 | 0.14 (0.095; 0.27) | 0.090 (0.060; 0.11) | 0.093 |
| 28:0 | 0.030 (0.022; 0.052) | 0.015 (0.010; 0.035) | 0.171 |
| **Total ECFA** | **39.9 (38.3; 43.4)** | **37.6 (36.4; 38.3)** | **0.065** |
| 15:0 | 1.04 (0.58; 1.54) | 0.61 (0.50; 0.71) | 0.093 |
| 17:0 | 0.49 (0.38; 0.66) | 0.40 (0.32; 0.42) | 0.127 |
| 19:0 | 0.15 (0.12; 0.20) | 0.15 (0.13; 0.18) | 1.000 |
| 21:0 | 0.10 (0.072; 0.16) | 0.070 (0.050; 0.070) | 0.073 |
| 23:0 | 0.13 (0.10; 0.25) | 0.11 (0.085; 0.13) | 0.284 |
| 25:0 | 0.13 (0.095; 0.35) | 0.095 (0.065; 0.13) | 0.171 |
| **Total OCFA** | **2.02 (1.35; 3.14)** | **1.50 (1.14; 1.66)** | **0.171** |
| 2,6,10-triM-12:0 | 0.050 (0.025; 0.075) | 0.030 (0.030; 0.040) | 0.524 |
| 4,8,12-triM-14:0 | 0.080 (0.045; 0.12) | 0.040 (0.030; 0.040) | **0.030** |
| **Total triM BCFA** | **0.13 (0.070; 0.20)** | **0.070 (0.055; 0.088)** | **0.093** |
| 12-M-13:0 | 0.040 (0.015; 0.060) | 0.020 (0.012; 0.030) | 0.222 |
| 13-M-14:0 | 0.060 (0.025; 0.10) | 0.16 (0.15; 0.18) | **0.003** |
| 14-M-15:0 | 0.18 (0.13; 0.30) | 0.42 (0.34; 0.46) | **0.010** |
| 15-M-16;0 | 0.10 (0.090; 0.17) | 0.58 (0.57; 0.61) | **0.003** |
| 17-M-18:0 | 0.030 (0.010; 0.035) | 0.045 (0.032; 0.068) | **0.045** |
| 18-M-19:0 | 0.020 (0.020; 0.030) | 0.030 (0.020; 0.038) | 0.354 |
| 20-M-21:0 | 0.040 (0.020; 0.055) | 0.030 (0.012; 0.050) | 0.724 |
| 22-M-23:0 | 0.030 (0.015; 0.030) | 0.030 (0.022; 0.040) | 0.222 |
| **Total iso BCFA** | **0.51 (0.32; 0.77)** | **1.30 (1.20; 1.42)** | **0.006** |
| 12-M-14:0 | 0.16 (0.070; 0.30) | 0.065 (0.060; 0.10) | 0.171 |
| 14-M-16:0 | 0.030 (0.020; 0.065) | 0.020 (0.020; 0.030) | 0.202 |
| 16-M-18:0 | 0.050 (0.035; 0.12) | 0.060 (0.020; 0.092) | 0.662 |
| 20-M-22:0 | 0.050 (0.020; 0.085) | 0.020 (0.010; 0.020) | **0.045** |
| 22-M-24:0 | 0.070 (0.030; 0.11) | 0.020 (0.012; 0.030) | **0.019** |
| **Total anteiso BCFA** | **0.30 (0.16; 0.40)** | **0.22 (0.20; 0.30)** | **0.556** |
| **Total BCFA** | **1.04 (0.58; 1.62)** | **1.52 (1.46; 1.72)** | **0.171** |
| **Total SFA** | **42.7 (40.4; 48.2)** | **40.4 (39.0; 41.6)** | **0.093** |
| 14:1 | 0.26 (0.15; 0.33) | 0.16 (0.13; 0.19) | 0.109 |
| 15:1 | 0.18 (0.12; 0.30) | 0.080 (0.052; 0.098) | **0.008** |
| 16:1 | 5.16 (3.68; 6.62) | 4.96 (4.32; 5.04) | 0.808 |
| 17:1 | 0.12 (0.062; 0.18) | 0.080 (0.070; 0.10) | 0.683 |
| 18:1 | 21.3 (19.7; 21.7) | 23.2 (20.9; 25.1) | 0.127 |
| 20:1 | 0.38 (0.22; 0.57) | 0.58 (0.45; 0.67) | 0.093 |
| 22:1 | 0.18 (0.11; 0.35) | 0.13 (0.10; 0.14) | 0.202 |
| 24:1 | 0.72 (0.32; 0.78) | 0.94 (0.52; 1.07) | 0.202 |
| **Total MUFA** | **29.2 (26.4; 30.0)** | **29.6 (27.5; 31.9)** | **0.435** |
| LA (18:2 n-6) | 15.7 (11.7; 17.8) | 16.8 (16.3; 18.1) | 0.127 |
| ARA (20:4 n-6) | 7.53 (6.33; 9.22) | 7.85 (6.82; 9.15) | 1.000 |
| DGLA (20:3 n-6) | 1.03 (0.69; 1.30) | 1.08 (0.88; 1.23) | 0.943 |
| EDA (20:2 n-6) | 0.52 (0.30; 0.73) | 0.46 (0.36; 0.51) | 0.354 |
| AdA (22:4 n-6) | 2.15 (1.38; 2.60) | 2.02 (1.60; 2.31) | 0.724 |
| **Total PUFA n-6** | **26.7 (20.6; 31.6)** | **28.3 (28.0; 29.6)** | **0.435** |
| ALA (18:3 n-3) | 0.12 (0.095; 0.22) | 0.095 (0.090; 0.10) | 0.065 |
| EPA (20:5 n-3) | 0.060 (0.040; 0.075) | 0.075 (0.060; 0.080) | 0.354 |
| DHA (22:6 n-3) | 1.08 (0.80; 1.31) | 1.12 (1.01; 1.28) | 0.724 |
| DPA n-3 (22:5 n-3) | 0.14 (0.11; 0.16) | 0.15 (0.14; 0.19) | 0.202 |
| **PUFA n-3** | **1.40 (1.21; 1.60)** | **1.44 (1.32; 1.64)** | **0.833** |
| **Supplemented iso-BCFA** | 0.35 (0.24; 0.56) | 1.14 (1.06; 1.24) | **0.030** |
| **Others iso-BCFA** | 0.16 (0.080; 0.21) | 0.16 (0.13; 0.19) | 0.622 |
| **C18:1/C18:0 ratio (SCD1)** | 1.92 (1.65; 1.98) | 2.16 (1.93; 2.57) | 0.093 |
| **ARA/DGLA ratio (D5D)** | 7.56 (6.93; 9.58) | 7.46 (7.20; 1.94) | 0.833 |
| **AdA/ARA ratio** | 0.24 (0.20; 0.32) | 0.24 (0.24; 0.27) | 0.833 |
| **ALA/LA ratio (D6D)** | 0.008 (0.005; 0.020) | 0.005 (0.005; 0.006) | 0.127 |
| **C22:0/C20:0 ratio (Elovl1)** | 1.09 (0.94; 1.20) | 0.93 (0.83; 1.00) | 0.065 |
| **C18:0/C16:0 ratio (Elovl6)** | 0.46 (0.44; 0.49) | 0.46 (0.44; 0.49) | 0.943 |
| **DHA/DPA n-3 ratio** | 7.71 (5.85; 11.2) | 7.67 (6.55; 7.79) | 0.530 |
| **ARA/LA ratio (Elovl5)** | 0.51 (0.49; 0.57) | 0.46 (0.37; 0.57) | 0.354 |

Value mediana (Q1; Q3); p value from non-parametric Mann-Whitney test on rank; AdA – adrenic acid, ALA - alpha-linolenic acid, ARA – arachidonic acid, BCFA – branched chain fatty acids, D5D - delta-5 desaturase; D6D - delta-6 desaturase  DGLA – dihomo-gamma-linolenic acid, DHA – docosahexaenoic acid, DPA – docosapentaenoic acid, ECFA – even chain fatty acids, EDA – eicosadienoic acid, ELOVL – elongase of fatty acid, EPA – eicosapentaenoic acid, ETA – eicosapentatetranoic acid, HDA – hexadecadienoic acid, LA – linoleic fatty acid, MUFA – monounsaturated fatty acids, OCFA – odd chain fatty acids, PUFA – polyunsaturated fatty acids, SCD1 - stearoyl-CoA desaturase 1; SFA – saturated fatty acids. Bold represents main groups of fatty acids.

Table S3: Content (% of total FA) of FA in the heart of 12-month-old SD-fed and BCFA-fed mice

| **FA** | **SD-fed mice** | **BCFA-fed mice** | **p value** |
| --- | --- | --- | --- |
| 10:0 | traces | traces | - |
| 12:0 | 0.020 (0.015; 0.020) | 0.030 (0.020; 0.040) | 0.149 |
| 14:0 | 0.27 (0.21; 0.40) | 0.33 (0.20; 0.47) | 0.808 |
| 16:0 | 13.0 (12.5; 14.3) | 13.2 (12.5; 15.2) | 0.933 |
| 18:0 | 17.2 (13.7; 17.9) | 17.3 (16.4; 19.1) | 0.435 |
| 20:0 | 0.41 (0.37; 0.44) | 0.32 (0.30; 0.36) | 0.065 |
| 22:0 | 0.16 (0.14; 0.19) | 0.14 (0.11; 0.16) | 0.127 |
| 24:0 | 0.10 (0.10; 0.16) | 0.095 (0.080; 0.12) | 0.354 |
| 26:0 | 0.010 (0.010; 0.010) | 0.010 (0.010; 0.010) | 1.000 |
| **Total ECFA** | **31.8 (30.8; 32.6)** | **31.7 (30.9; 32.6)** | 0.833 |
| 11:0 | traces | traces | - |
| 13:0 | traces | traces | - |
| 15:0 | 0.080 (0.075; 0.11) | 0.11 (0.082; 0.14) | 0.171 |
| 17:0 | 0.29 (0.24; 0.30) | 0.30 (0.27; 0.32) | 0.435 |
| 19:0 | 0.30 (0.16; 0.31) | 0.30 (0.28; 0.32) | 0.622 |
| 21:0 | 0.020 (0.015; 0.035) | 0.025 (0.020; 0.030) | 0.724 |
| 23:0 | 0.040 (0.025; 0.040) | 0.035 (0.030; 0.040) | 0.833 |
| 25:0 | 0.010 (0.010; 0.010) | 0.010 (0.010; 0.010) | 1.000 |
| **Total OCFA** | **0.74 (0.56; 0.78)** | **0.76 (0.72; 0.84)** | **0.284** |
| 2,5,10-triM-12:0 | 0.010 (0.010; 0.010) | 0.010 (0.010; 0.010) | 0.755 |
| 4,8,12-triM-14:0 | 0.010 (0.010; 0.010) | 0.010 (0.010; 0.010) | 1.000 |
| **Total triM BCFA** | **0.010 (0.010; 0.015)** | **0.020 (0.020; 0.020)** | **0.048** |
| 12-M-13:0 | traces | traces | - |
| 13-M-14:0 | 0.010 (0.010; 0.020) | 0.060 (0.040; 0.070) | **0.002** |
| 14-M-15:0 | 0.040 (0.035; 0.070) | 0.20 (0.17; 0.25) | **0.002** |
| 15-M-16:0 | 0.15 (0.10; 0.16) | 0.48 (0.46; 0.53) | **0.002** |
| 16-M-17:0 | 0.070 (0.055; 0.080) | 0.12 (0.090; 0.16) | **0.003** |
| 17-M-18:0 | 0.040 (0.030; 0.045) | 0.070 (0.062; 0.078) | **0.002** |
| 18-M-19:0 | 0.020 (0.020; 0.025) | 0.020 (0.020; 0.020) | 0.833 |
| 20-M-21:0 | 0.010 (0.010; 0.010) | 0.010 (0.010; 0.010) | 1.000 |
| **Total iso BCFA** | **0.34 (0.28; 0.38)** | **0.95 (0.93; 1.05)** | **0.002** |
| 10-M-12:0 | traces | traces | - |
| 12-M-14:0 | 0.010 (0.010; 0.010) | 0.015 (0.010; 0.028) | 0.171 |
| 14-M-16:0 | 0.010 (0.010; 0.010) | 0.010 (0.010; 0.010) | 1.000 |
| 16-M-18:0 | 0.030 (0.020; 0.030) | 0.030 (0.020; 0.040) | 0.622 |
| 20-M-22:0 | 0.010 (0.010; 0.010) | 0.010 (0.010; 0.010) | 1.000 |
| 22-M-24:0 | traces | traces | - |
| **Total anteiso BCFA** | **0.050 (0.040; 0.055)** | **0.060 (0.038; 0.070)** | **0.171** |
| **Total BCFA** | **0.39 (0.34; 0.44)** | **1.04 (1.01; 1.10)** | **0.002** |
| **Total SFA** | **32.8 (32.0; 33.6)** | **33.5 (32.7; 34.4)** | **0.127** |
| 14:1 | 0.010 (0.010; 0.015) | 0.010 (0.010; 0.020) | 0.530 |
| 16:1 | 0.90 (0.71; 1.74) | 1.00 (0.72; 1.29) | 0.788 |
| 17:1 | 0.020 (0.015; 0.035) | 0.030 (0.030; 0.048) | 0.222 |
| 18:1 | 11.9 (11.3; 19.9) | 12.8 (11.3; 15.0) | 0.943 |
| 20:1 | 0.71 (0.51; 0.77) | 0.68 (0.56; 0.79) | 1.000 |
| 22:1 | 0.050 (0.035; 0.055) | 0.030 (0.030; 0.040) | 0.073 |
| 24:1 | 0.14 (0.078; 0.18) | 0.065 (0.040; 0.090) | **0.048** |
| **Total MUFA** | **13.7 (13.0; 21.6)** | **14.7 (13.1; 17.5)** | **0.724** |
| HDA (16:2 n-6) | 0.030 (0.025; 0.030) | 0.020 (0.012; 0.020) | **0.011** |
| LA (18:2 n-6) | 22.6 (22.1; 23.1) | 21.1 (20.5; 21.6) | **0.002** |
| ARA (20:4 n-6) | 11.3 (8.96; 11.9) | 10.7 (9.52; 12.2) | 0.724 |
| DGLA (20:3 n-6) | 0.63 (0.48; 0.68) | 0.63 (0.52; 0.70) | 0.833 |
| DPA n-6 (22:5 n-6) | 6.53 (3.76; 8.46) | 6.96 (6.52; 9.00) | 0.354 |
| AdA (22:4 n-6) | 1.45 (1.10; 1.50) | 1.35 (1.12; 1.40) | 0.284 |
| **Total PUFA n-6** | **44.1 (36.7; 44.6)** | **42.1 (39.3; 43.5)** | **0.354** |
| ALA (18:3 n-3) | 0.040 (0.040; 0.050) | 0.030 (0.020; 0.040) | 0.065 |
| EPA (20:5 n-3) | 0.060 (0.045; 0.065) | 0.060 (0.052; 0.070) | 0.524 |
| DHA (22:6 n-3) | 8.03 (5.70; 8.86) | 8.56 (6.55; 9.52) | 0.524 |
| DPA n-3 (22:5 n-3) | 0.48 (0.32; 0.59) | 0.48 (0.42; 0.52) | 1.000 |
| **Total PUFA n-3** | **8.69 (6.11; 9.52)** | **9.14 (7.08; 10.1)** | **0.724** |
| **Supplemented iso-BCFA** | 0.21 (0.16; 0.24) | 0.76 (0.68; 0.84) | **0.002** |
| **Others iso-BCFA** | 0.13 (0.11; 0.15) | 0.21 (0.19; 0.28) | **0.002** |
| **C18:1/C18:0 ratio (SCD1)** | 0.69 (0.63; 1.49) | 0.69 (0.58; 0.91) | 0.524 |
| **ARA/DGLA ratio (D5D)** | 0.057 (0.054; 0.058) | 0.056 (0.048; 0.064) | 0.833 |
| **AdA/ARA ratio** | 0.12 (0.10; 0.13) | 0.13 (0.12; 0.13) | 0.284 |
| **ALA/LA ratio (D6D)** | 0.0016 (0.0009; 0.0019) | 0.0018 (0.0018; 0.0022 | 0.222 |
| **C22:0/C20:0 ratio (Elovl1)** | 0.42 (0.38; 0.44) | 0.42 (0.34; 0.48) | 0.833 |
| **C18:0/C16:0 ratio (Elovl6)** | 1.30 (0.82; 1.42) | 1.37 (1.11; 1.42) | 0.435 |
| **DHA/DPA n-3 ratio** | 0.0075 (0.0067; 0.0090) | 0.0070 (0.0053; 0.0092) | 0.622 |
| **ARA/LA ratio (Elovl5)** | 0.50 (0.40; 0.52) | 0.51 (0.47; 0.56) | 0.435 |

Value mediana (Q1; Q3); p value from non-parametric Mann-Whitney test on rank; AdA – adrenic acid, ALA - alpha-linolenic acid, ARA – arachidonic acid, BCFA – branched chain fatty acids, D5D - delta-5 desaturase; D6D - delta-6 desaturase  DGLA – dihomo-gamma-linolenic acid, DHA – docosahexaenoic acid, DPA – docosapentaenoic acid, ECFA – even chain fatty acids, EDA – eicosadienoic acid, ELOVL – elongase of fatty acid, EPA – eicosapentaenoic acid, ETA – eicosapentatetranoic acid, HDA – hexadecadienoic acid, LA – linoleic fatty acid, MUFA – monounsaturated fatty acids, OCFA – odd chain fatty acids, PUFA – polyunsaturated fatty acids, SCD1 - stearoyl-CoA desaturase 1; SFA – saturated fatty acids. Bold represents main groups of fatty acids.

Table S4: Content (% of total FA) of FA in the serum of 12-month-old SD-fed and BCFA-fed mice.

| **FA** | **SD-fed mice** | **BCFA-fed mice** | **p value** |
| --- | --- | --- | --- |
| 12:0 | 0.050 (0.045; 0.065) | 0.040 (0.040; 0.050) | 0.284 |
| 14:0 | 0.36 (0.34; 0.49) | 0.55 (0.43; 0.68) | 0.093 |
| 16:0 | 16.4 (16.0; 18.1) | 16.5 (16.3; 17.1) | 0.943 |
| 18:0 | 7.00 (6.85; 7.26) | 7.22 (6.46; 7.59) | 0.833 |
| 20:0 | 0.46 (0.41; 0.60) | 0.46 (0.39; 0.50) | 0.724 |
| 22:0 | 0.37 (0.30; 0.44) | 0.39 (0.35; 0.41) | 0.524 |
| 24:0 | 0.30 (0.28; 0.36) | 0.36 (0.33; 0.43) | 0.127 |
| 26:0 | 0.030 (0.020; 0.040) | 0.030 (0.022; 0.045) | 0.943 |
| **Total ECFA** | **25.6 (24.4; 26.9)** | **25.7 (24.9; 26.1)** | **0.943** |
| 13:0 | 0.010 (0.010; 0.025) | 0.020 (0.020; 0.020) | 0.432 |
| 15:0 | 0.22 (0.18; 0.32) | 0.33 (0.30; 0.38) | 0.171 |
| 17:0 | 0.33 (0.30; 0.38) | 0.38 (0.32; 0.41) | 0.222 |
| 19:0 | 0.17 (0.16; 0.22) | 0.20 (0.18; 0.22) | 0.435 |
| 21:0 | 0.050 (0.045; 0.060) | 0.055 (0.042; 0.078) | 0.622 |
| 23:0 | 0.090 (0.075; 0.11) | 0.11 (0.10; 0.13) | 0.127 |
| 25:0 | 0.020 (0.015; 0.040) | 0.030 (0.012; 0.030) | 0.943 |
| **Total OCFA** | **0.86 (0.81; 1.15)** | **1.12 (1.02; 1.29)** | **0.284** |
| 2,6,10-triM-12:0 | 0.030 (0.015; 0.030) | 0.040 (0.032; 0.040) | **0.030** |
| 4,8,12-triM-14:0 | 0.020 (0.015; 0.025) | 0.030 (0.020; 0.030) | 0.222 |
| **Total triM BCFA** | **0.050 (0.035; 0.050)** | **0.070 (0.052; 0.070)** | **0.045** |
| 12-M-13:0 | 0.010 (0.010; 0.010) | 0.010 (0.010; 0.018) | 0.570 |
| 13-M-14:0 | 0.040 (0.030; 0.065) | 0.28 (0.24; 0.33) | **0.002** |
| 14-M-15:0 | 0.10 (0.095; 0.14) | 0.58 (0.55; 0.64) | **0.002** |
| 15-M-16:0 | 0.21 (0.16; 0.32) | 1.46 (1.32; 1.58) | **0.002** |
| 16-M-17:0 | 0.080 (0.065; 0.14) | 0.18 (0.18; 0.21) | **0.002** |
| 17-M-18:0 | 0.040 (0.035; 0.060) | 0.080 (0.062; 0.090) | **0.011** |
| 18-M-19:0 | 0.030 (0.030; 0.030) | 0.030 (0.030; 0.040) | 0.432 |
| 20-M-21:0 | 0.020 (0.010; 0.025) | 0.020 (0.020; 0.030) | 0.222 |
| 22-M-23:0 | 0.010 (0.010; 0.010) | 0.020 (0.010; 0.020) | **0.048** |
| **Total iso BCFA** | **0.52 (0.48; 0.78)** | **2.70 (2.49; 2.92)** | **0.002** |
| 12-M-14:0 | 0.050 (0.030; 0.080) | 0.070 (0.052; 0.088) | 0.354 |
| 14-M-16:0 | 0.040 (0.040; 0.045) | 0.070 (0.050; 0.070) | **0.019** |
| 16-M-18:0 | 0.020 (0.020; 0.020) | 0.020 (0.020; 0.030) | 0.524 |
| 20-M-22:0 | 0.020 (0.010; 0.030) | 0.025 (0.020; 0.030) | 0.524 |
| 22-M-24:0 | 0.010 (0.000; 0.010) | 0.010 (0.010; 0.015) | 0.222 |
| **Total anteiso BCFA** | **0.14 (0.11; 0.19)** | **0.22 (0.19; 0.24)** | **0.065** |
| **Total BCFA** | **0.71 (0.62; 1.02)** | **3.00 (2.72; 3.22)** | **0.002** |
| **Total SFA** | **27.5 (25.9; 28.9)** | **29.8 (28.8; 30.6)** | **0.030** |
| 14:1 | 0.080 (0.035; 0.10) | 0.11 (0.075; 0.13) | 0.222 |
| 15:1 | 0.040 (0.020; 0.070) | 0.075 (0.052; 0.10) | 0.127 |
| 16:1 | 2.92 (2.40; 3.17) | 3.16 (2.86; 5.54) | 0.222 |
| 17:1 | 0.050 (0.035; 0.070) | 0.095 (0.062; 0.12) | **0.030** |
| 18:1 | 23.0 (22.3; 24.7) | 22.9 (21.6; 25.6) | 0.943 |
| 20:1 | 1.40 (0.89; 1.48) | 1.22 (1.12; 1.48) | 0.622 |
| 22:1 | 0.090 (0.070; 0.14) | 0.11 (0.082; 1.18) | 0.524 |
| 24:1 | 0.22 (0.16; 0.24) | 0.23 (0.21; 0.26) | 0.435 |
| **Total MUFA** | **27.4 (26.3; 29.8)** | **28.2 (26.7; 33.2)** | **0.833** |
| HDA (16:2 n-6) | 0.030 (0.020; 0.045) | 0.030 (0.022; 0.030) | 0.524 |
| LA (18:2 n-6) | 32.5 (30.6; 33.0) | 29.1 (25.5; 30.2) | **0.003** |
| ARA (20:4 n-6) | 9.86 (8.52; 11.9) | 9.48 (8.92; 11.1) | 0.943 |
| DGLA (20:3 n-6) | 0.48 (0.38; 0.52) | 0.51 (0.44; 0.56) | 0.284 |
| EDA (20:2 n-6) | 0.080 (0.055; 0.10) | 0.085 (0.062; 0.10) | 0.833 |
| DPA n-6 (22:5 n-6) | 0.43 (0.36; 0.51) | 0.53 (0.40; 0.60) | 0.171 |
| AdA (22:4 n-6) | 0.18 (0.14; 0.22) | 0.18 (0.16; 0.20) | 0.943 |
| **Total PUFA n-6** | **43.5 (40.1; 46.2)** | **40.4 (35.4; 42.1)** | **0.127** |
| ALA (18:3 n-3) | 0.31 (0.26; 0.42) | 0.29 (0.24; 0.34) | 0.524 |
| EPA (20:5 n-3) | 0.11 (0.080; 0.13) | 0.11 (0.082; 0.12) | 1.000 |
| ETA (20:4 n-3) | 0.040 (0.035; 0.040) | 0.035 (0.030; 0.048) | 0.833 |
| DHA (22:6 n-3) | 0.87 (0.80; 1.02) | 0.91 (0.83; 1.08) | 0.724 |
| DPA n-3 (22:5 n-3) | 0.060 (0.050; 0.065) | 0.060 (0.042; 0.060) | 0.622 |
| **Total PUFA n-3** | **1.49 (1.24; 1.61)** | **1.42 (1.26; 1.62)** | **0.943** |
| **Supplemented iso-BCFA** | 0.34 (0.30; 0.52) | 2.30 (2.12; 2.54) | **0.002** |
| **Others iso-BCFA** | 0.21 (0.16; 0.26) | 0.36 (0.32; 0.40) | **0.002** |
| **C18:1/C18:0 ratio (SCD1)** | 3.28 (3.22; 3.44) | 3.07 (2.89; 4.05) | 0.724 |
| **ARA/DGLA ratio (D5D)** | 0.043 (0.042; 0.051) | 0.052 (0.042; 0.058) | 0.435 |
| **AdA/ARA ratio** | 0.018 (0.017; 0.019) | 0.018 (0.015; 0.022) | 0.943 |
| **ALA/LA ratio (D6D)** | 0.009 (0.008; 0.013) | 0.010 (0.009; 0.012) | 1.000 |
| **C22:0/C20:0 ratio (Elovl1)** | 0.76 (0.72; 0.78) | 0.85 (0.78; 0.86) | 0.019 |
| **C18:0/C16:0 ratio (Elovl6)** | 0.41 (0.40; 0.43) | 0.43 (0.40; 0.45) | 0.524 |
| **DHA/DPA n-3 ratio** | 15.4 (14.4; 17.1) | 16.2 (14.3; 21.8) | 0.622 |
| **ARA/LA ratio (Elovl5)** | 0.30 (0.28; 0.36) | 0.34 (0.33; 0.38) | 0.222 |

Value mediana (Q1; Q3); p value from non-parametric Mann-Whitney test on rank; AdA – adrenic acid, ALA - alpha-linolenic acid, ARA – arachidonic acid, BCFA – branched chain fatty acids, D5D - delta-5 desaturase; D6D - delta-6 desaturase  DGLA – dihomo-gamma-linolenic acid, DHA – docosahexaenoic acid, DPA – docosapentaenoic acid, ECFA – even chain fatty acids, EDA – eicosadienoic acid, ELOVL – elongase of fatty acid, EPA – eicosapentaenoic acid, ETA – eicosapentatetranoic acid, HDA – hexadecadienoic acid, LA – linoleic fatty acid, MUFA – monounsaturated fatty acids, OCFA – odd chain fatty acids, PUFA – polyunsaturated fatty acids, SCD1 - stearoyl-CoA desaturase 1; SFA – saturated fatty acids. Bold represents main groups of fatty acids.


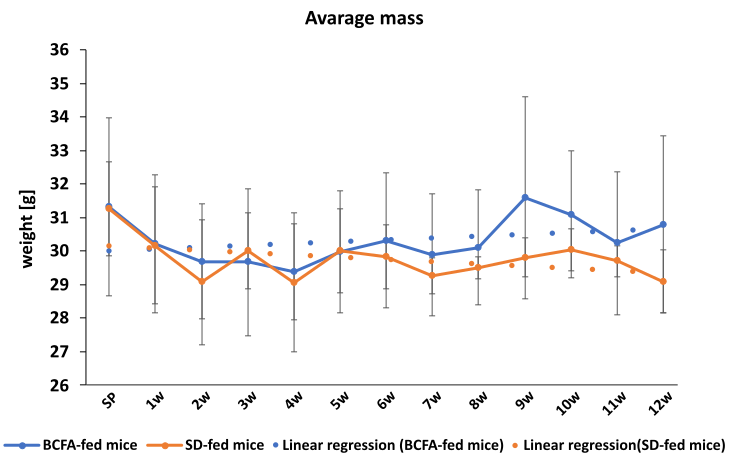


Figure S1: The body weight in BCFA-fed and SD-fed mice measured week by week. The data show mean ± SD. SP – the beginning of the experiment (first body weight measured), w – week.


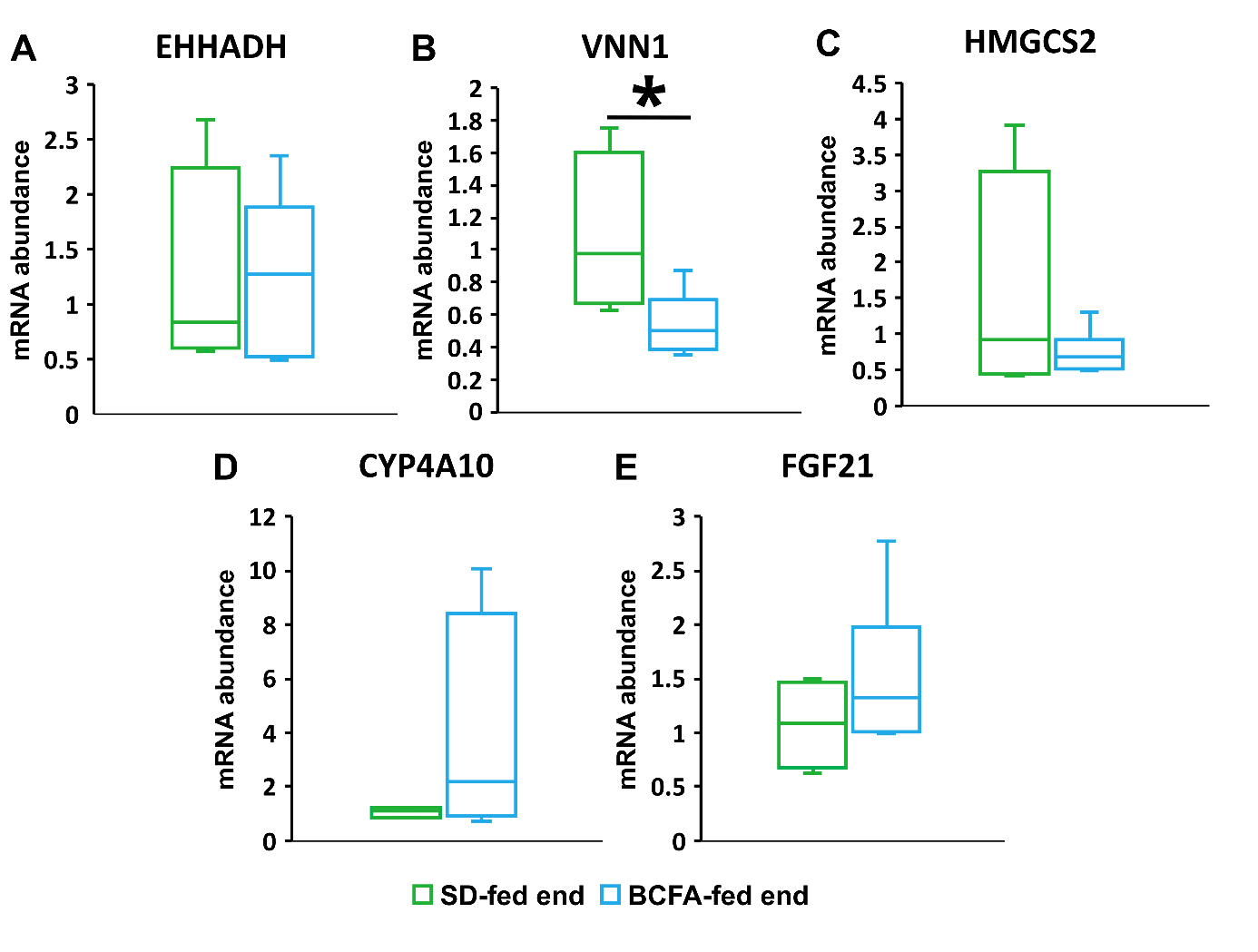


Figure S2: mRNA relative levels of selected genes regulated by PPARa in the livers from BCFA-fed and SD-fed *ApoE^-/-^/Ldlr^⁻/⁻^* mice: (A) *Ehhadh*, (B) *Vnn1*, (C) *Hmgcs2*, (D) *Cyp4a10*, (E) *Fgf21.*  *p < 0.05, p value from two-sample t-tests, followed by Mann-Whitney rank-sum test.
